# Malignant transformation of a phalangeal enchondroma into a recurrent grade II chondrosarcoma requiring successive transcarpal amputations: a case report

**DOI:** 10.1080/23320885.2022.2099864

**Published:** 2022-07-19

**Authors:** Ceyran Hamoudi, Benjamin Bouillet, Antoine Martins

**Affiliations:** a Department of Hand Surgery, SOS main, University hospital of Strasbourg, Strasbourg, France; b Hand, Peripheral Nerves, and Microsurgery Unit, SOS Main Auvergne, La Chataigneraie Hospital, Beaumont, France

**Keywords:** Enchondroma, chondrosarcoma, hand, malignant transformation, small bone tumor, posterior interosseous artery flap, transcarpal amputation

## Abstract

We report a case of malignant transformation of a phalangeal enchondroma into a grade II chondrosarcoma requiring two successive transcarpal amputations owing to recurrence. Soft tissue defects were repaired using single-stage reconstruction with a posterior interosseous artery flap. The 2-year follow-up assessment was satisfactory and no recurrence was observed.

## Introduction

Chondrosarcomas are the most common primary malignant bone tumor except for osteosarcomas [1]. They represent 20%–27% of all primary bone tumors and their occurrence is equally distributed with respect to gender, with a reported incidence of 1:200,000–1:500,000 [2–4].

Sites where they commonly occur are the pelvis, proximal femur, and proximal humerus in patients aged between 50 and 70 years [3]. Hand chondrosarcomas are rare, accounting for 1.5%–3.2% of all chondrosarcomas [5,6]. They usually involve the phalanges, metacarpals, trapezium, and trapezoid [7].

These tumors are composed of cartilage-producing cells and usually present as slow-growing and painful masses. They are histologically classified into three grades: grade I or low, grade II or intermediate, and grade III or high [8], with the majority of lesions belonging to the low-grade category [9]. Oncological outcomes are mainly determined by histological grading: the higher the grade, the more likely it is that the tumor will metastasize [4].

Although they may be primary tumors, chondrosarcomas can also result from the malignant transformation of a pre-existing lesion, such as enchondromas, multiple enchondromatosis, or osteochondromas [6,7,10]. Benign solitary enchondromas are the most common tumors found in the hand and are usually located in the phalanges [11]. It is difficult to differentiate between enchondromas and low-grade chondrosarcomas (LGCS) because of their similar appearance on radiography and biopsy [12]. Intermediate- and high-grade chondrosarcomas demonstrate more specific signs, such as perilesional edema and cortical destruction. LGCS are usually treated by means of amputation, however, more recent literature advocates for intralesional surgery [4]. Wide resection is usually recommended to avoid recurrence, as adjuvant therapies are ineffective [13].

It is particularly challenging to manage soft tissue defects of the hand following wide-margin resection because numerous underlying structures such as bones, tendons, vascular pedicles, and nerves are exposed. Various types of hand reconstruction techniques and skin flaps have been described, to preserve limb function as well as the integrity of underlying structures; however, the posterior interosseous artery (PIA) flap is the option that was selected by us. These flaps are known to be reliable for wrist and hand reconstruction in the background of traumatic soft tissue defects [14] and following resection of soft tissue [15].

In this case report, we describe a rare case of a patient initially treated with curettage and bone filling for a symptomatic enchondroma of the fourth proximal phalanx that presented with malignant transformation to secondary chondrosarcoma (Grade II) at the first follow-up at one year following initial treatment. We performed a wide resection of the 4th ray, and a recurrence of the lesion was discovered again at the 8-month follow-up, which resulted in ulnar transcarpal amputation. The hand defect was repaired using a posterior interosseous artery flap, and at the follow-up 2 years after transcarpal amputation, the clinical evaluation was satisfactory.

## Case report

A 66-year-old Caucasian man with no significant medical history was referred to our department of hand surgery and treated for an enchondroma of the proximal phalanx of the fourth ray of the left hand with curettage and bone filling using a cancellous allograft. Anatomopathological analysis revealed an enchondroma without malignancy.

**Figure 1.**
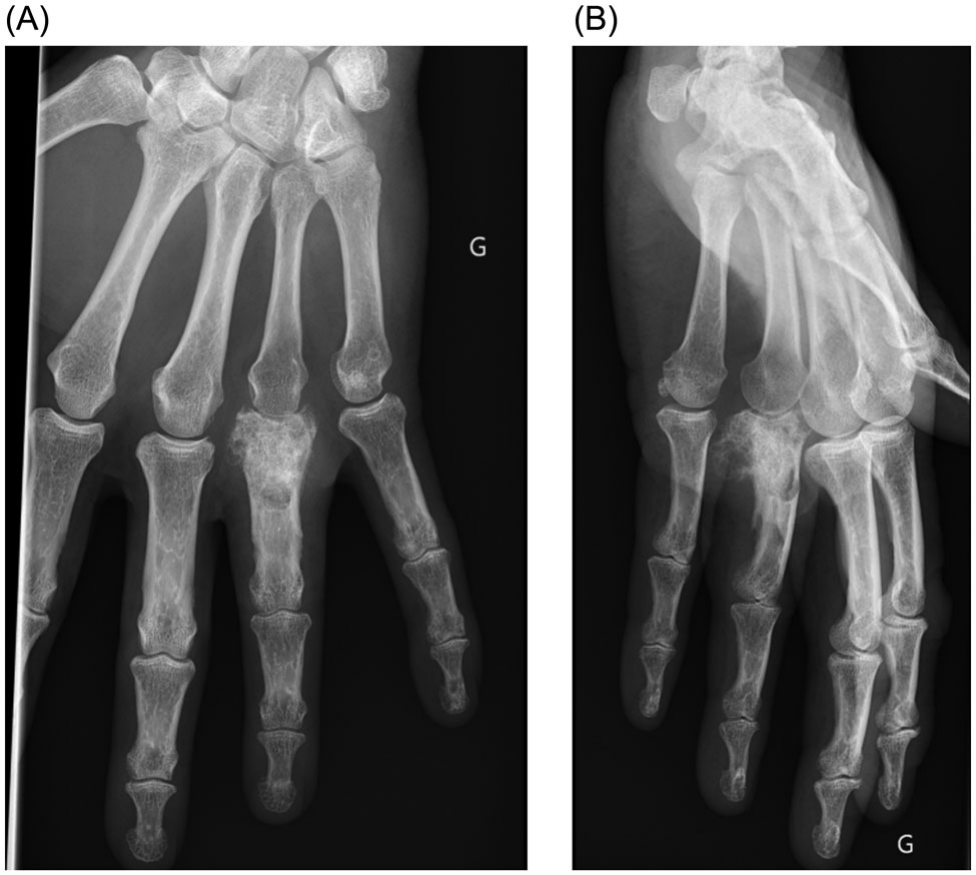
(A) Plain AP view of the hand, bone lysis and periosteal reaction are visible on the proximal phalanx of the fourth ray. G Left side (in French: Gauche). (B) Plain lateral view of the hand. G Left side (in French: Gauche)

**Figure 2.**
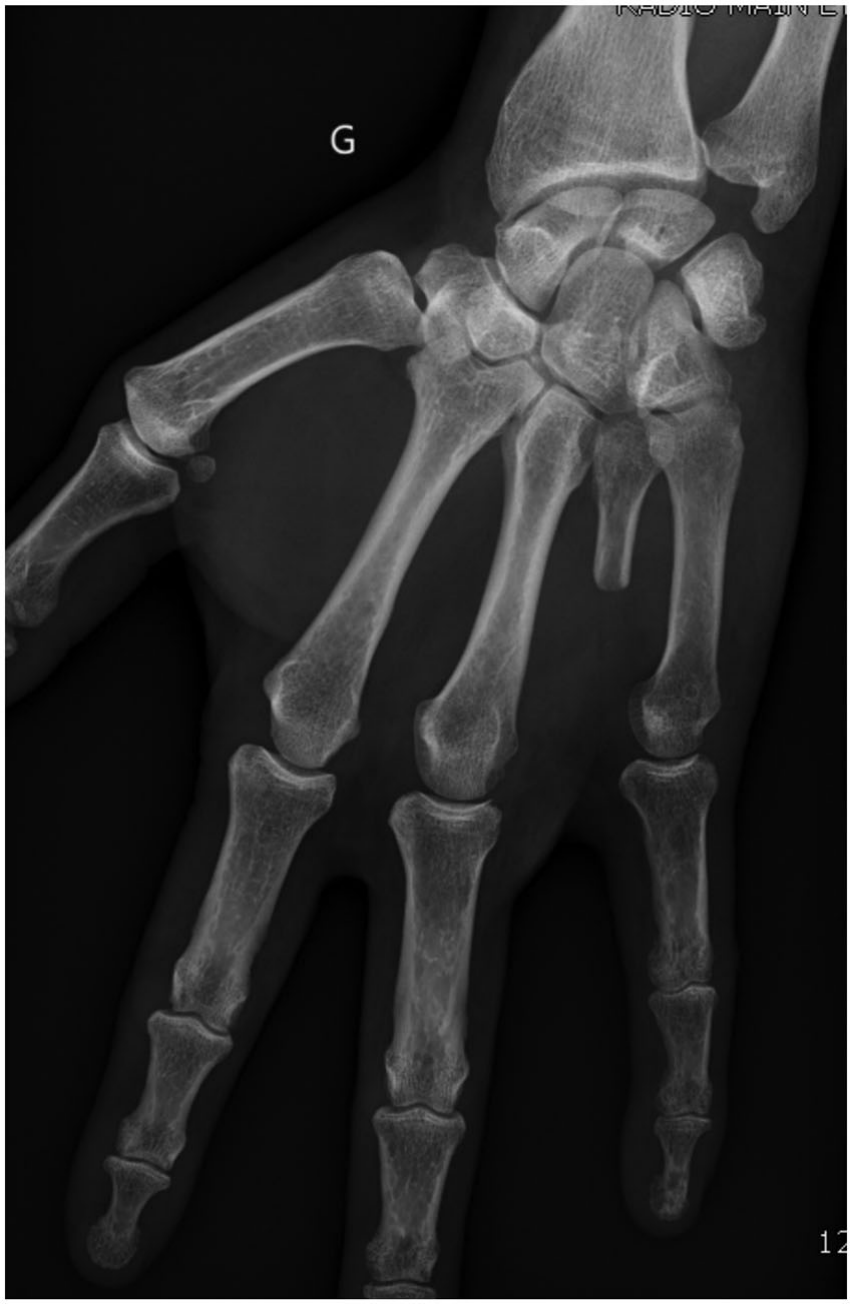
Plain AP view of the hand after amputation of the fourth ray, as per the modified Le Viet technique. G Left side (in French: Gauche).

A year later, the patient presented with a mass appearing next to the metacarpophalangeal joint on the fourth ray, along with persistent pain. Standard radiographs revealed bone lysis and periosteal reaction suggestive of malignant evolution of the previous lesion’s remnants into chondrosarcoma (Figures 1a and b). Magnetic resonance imaging (MRI) was also performed to determine the extent of the tumor. Biopsy confirmed the presence of a grade 2 chondrosarcoma. The patient then underwent tumor resection with amputation of the fourth ray at the base of the metacarpal, as per the modified Le Viet technique (Figure 2). The resected specimen was sent for pathological analysis to confirm chondrosarcoma and determine appropriate surgical margins.

**Figure 3.**
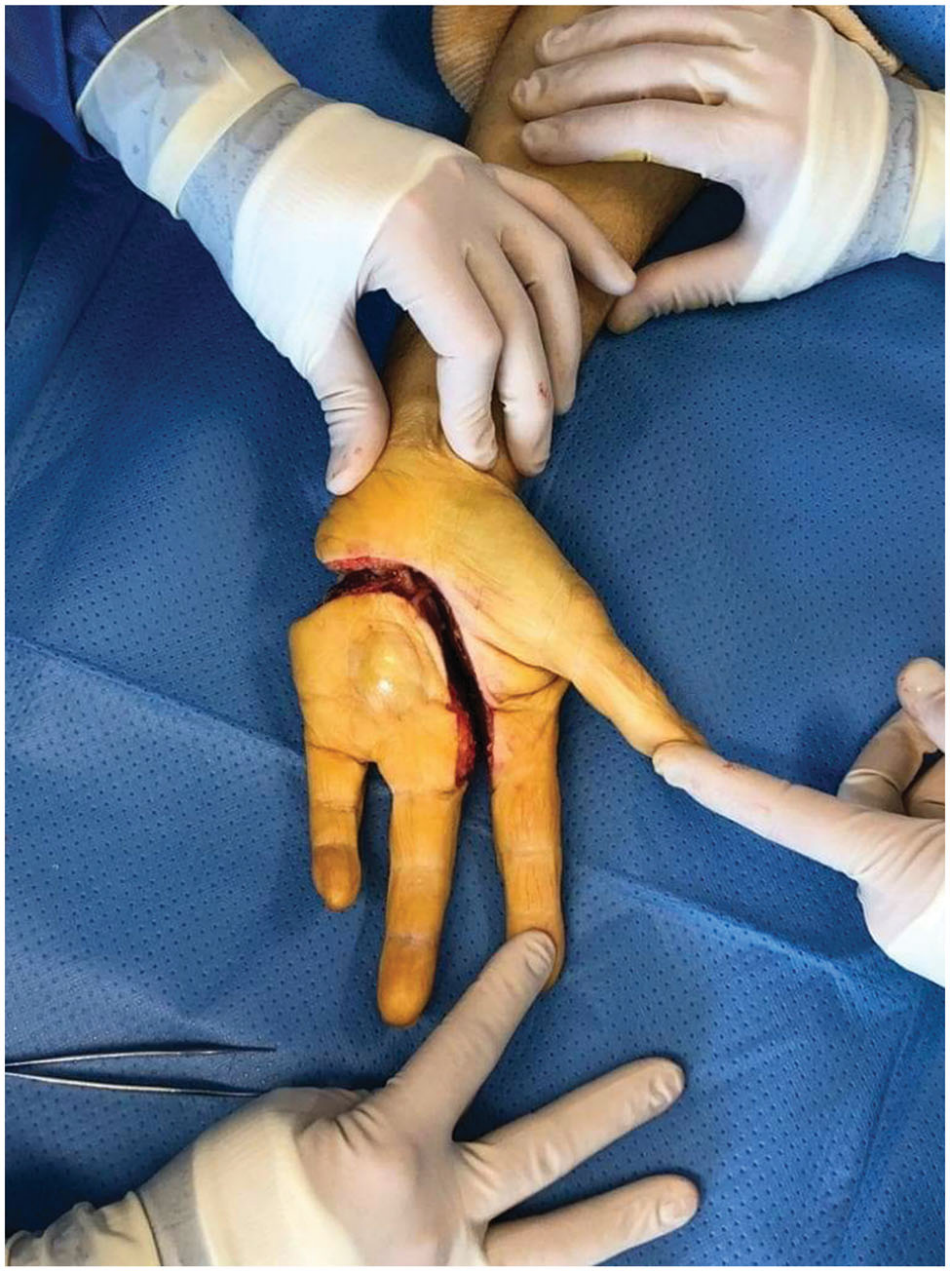
Palmar view of the hand after transcarpal amputation.

**Figure 4.**
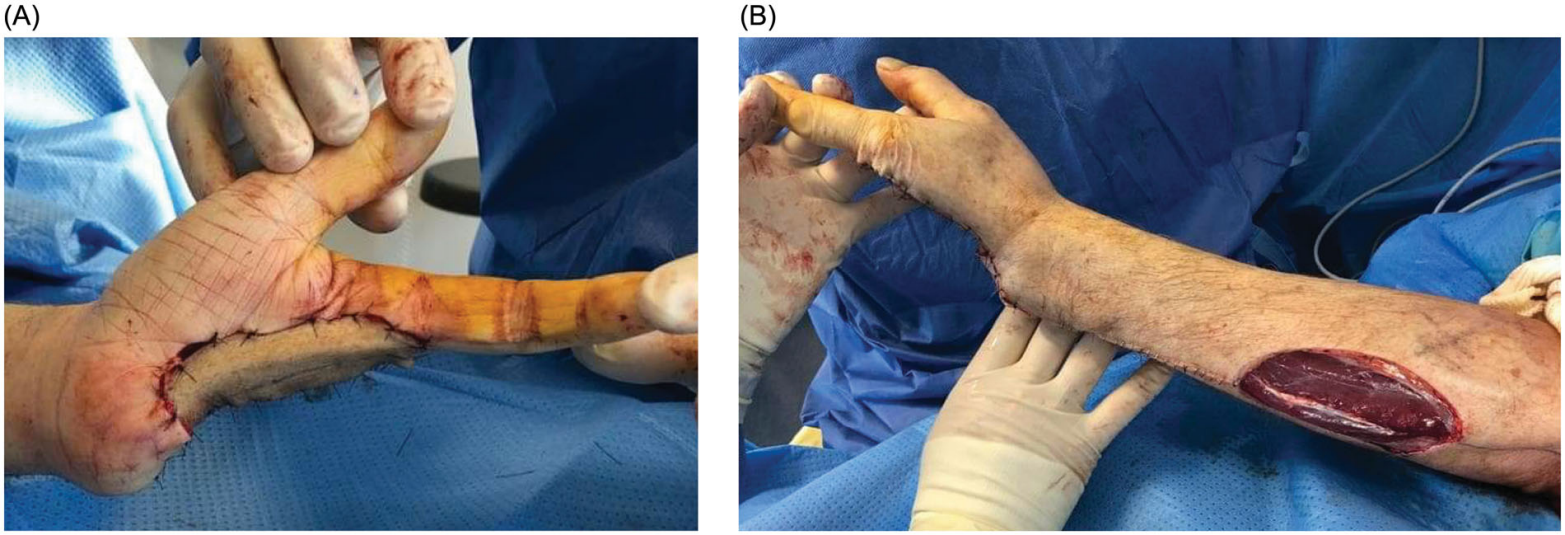
(A) Palmar view of the hand after the defect was covered with a posterior interosseous flap. (B) Dorsal view of the forearm with the donor site.

Unfortunately, recurrence was observed 8 months following the resection. Local revision surgery was performed, prior to which the patient was informed of the intraoperative risk of amputation being required in the forearm. Transcarpal amputation was performed, and only the first two rays of the hand were preserved (Figure 3). The motor branch of the ulnar nerve was preserved. To avoid instability and radialization of the wrist, the extensor carpi ulnaris tendon was preserved and fixed to the triquetrum using an anchor (Non-absorbable Mitek© 3.0). The defect was covered with a posterior interosseous flap (Figures 4a and b).

**Figure 5.**
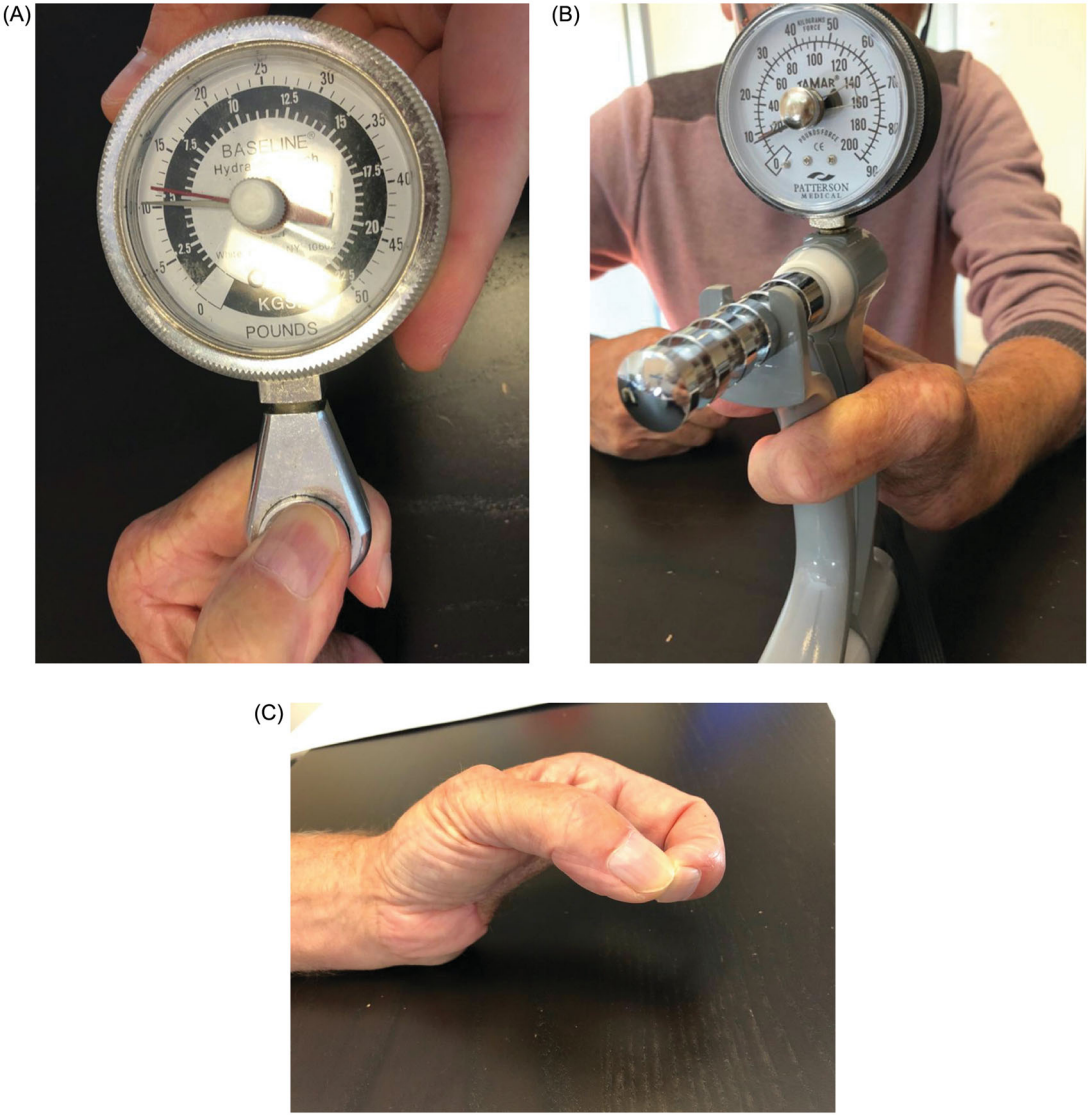
(A) The key pinch test with a thumb-index force at 5.5 kg. (B) The Jamar hand dynamometer with a grip force of 10 kg. (C) The pincer grasp of the patient.

The evolution of the flap was favorable. Two years later, the patient showed satisfactory functional outcomes, without any signs of local or distant recurrence. No complications occurred at the donor sites. The patient reported no hand or wrist pain at the last follow-up. The thumb-index force was 5.5 kg and the wrist was capable of generating a force of 10 kg (Figure 5a–c). The patient was able to perform most activities of daily living by this juncture.

## Discussion and conclusions

Asymptomatic enchondromas are usually incidental findings that do not require immediate treatment. On radiographs, they are intramedullary lucent lesions with lobulated contour, endosteal scalloping, and chondroid calcification [16]. Enchondromas can simply be observed, and radiographic follow-up is not required. However, when symptoms such as pain, swelling, deformity, or pathological fractures are present, surgical treatment is recommended [17].

Moreover, pain in the absence of a pathological fracture can be an important feature to differentiate LGCS from enchondromas, which are usually painless because of their slow growth, minimal peritumoral reaction, and avascularity [6]. This differentiation is essential in the selection of an appropriate treatment modality and for decreasing the rate of recurrence. First-line treatment of enchondromas comprises of intralesional curettage and filling of the defect using autologous or allogenic bone grafting or augmentation [17]. Single-stage curettage is usually preferred over delayed surgery [18,19]. Another possible surgical procedure that may be indicated is additional plate fixation to reduce the risk of nonunion of displaced pathological fractures [20]. To decrease the rate of recurrence and eliminate residual tumor cells, surgical adjuncts such as bone cement can be used [21]. In our case, defect filling following curettage was performed by augmentation using a cancellous allograft.

Recurrence of enchondromas is uncommon, and malignant transformation of a benign enchondroma or multiple enchondromatosis to secondary chondrosarcomas in the hand is extremely rare [6]. Some conditions, such as Ollier disease or Maffucci syndrome, characterized by multiple enchondromas, are associated with a high malignant transformation rate, varying from 20% to 57% [10]. Nevertheless, in our case, a malignant transformation of an enchondroma to a grade II chondrosarcoma was observed.

Our case report highlights the difficulty in differentiating between enchondromas and low-grade chondrosarcomas at the time of initial diagnosis. When in doubt, apart from standard clinical as well as radiological evaluations, MRI and histopathological evaluations should be performed. In our patient, the biopsy revealed a benign enchondroma without histological features suggestive of malignancy. Welkerling et al. reported that the same biopsy can be graded differently by different labs [22]. This caveat must be kept in mind by hand surgeons, who will inevitably treat many enchondromas during the course of their careers.

Chondrosarcomas vary from low-grade, which are relatively benign to high-grade, or dedifferentiated tumors which are associated with very poor survival rates [4]. On the hand, primary chondrosarcomas are rare entities, found in phalangeal bones in 60% of cases and in the metacarpals in the remaining 40% of cases [23]. Radiography shows intralesional calcification with the popcorn-like pattern, cortical erosion, periosteal reaction, and soft tissue mass [16]. An LGCS of the hand behaves as a locally aggressive lesion and, in contrast to chondrosarcomas located in the talus and calcaneus, rarely metastasizes [13]. Nevertheless, pulmonary metastasis has been reported in higher-grade tumors [6]. Historically, LGCS are treated by wide-margin resection, however, recently there has been a tendency to perform intralesional surgery with curettage, along with the concurrent use of local adjuvants, such as phenol, bone polymethyl methacrylate and cryotherapy [4]. Dierselhuis et al. reviewed 18 studies and concluded that intralesional treatment is associated with better functional outcomes and equal recurrence-free survival compared to wide-margin resection [4]. In grade II chondrosarcomas, as in our case, wide resection of local healthy tissue is required, to avoid local recurrence or metastases [24]. We first performed a trans-metacarpal osteotomy of the fourth ray, which was as per a modified version of the original Le Viet technique [25,26]. Following the diagnosis of the second recurrence, we performed a partial amputation of the fifth and third rays, which resulted in a large soft-tissue defect. In similar cases, hand amputation is also a viable treatment option [5].

In large soft tissue defects, the primary focus is to minimize donor site morbidity and at the same time, to obtain sufficient flap coverage to preserve limb function. In our case, we chose a PIA flap as a therapeutic option for reconstruction. It was first described as a reverse-flow pedicle flap in 1986 [27–30] and provides a reliable flap with adequate vasculature. to cover wrist and hand defects [31]. The PIA flap does not sacrifice the major blood vessels of the hand because it relies on the posterior interosseous artery and its anastomosis with the anterior interosseous artery, which offers a much-needed alternative option, when the radial or ulnar artery is damaged or when palmar arches are absent. Jones et al. reported that the reverse radial forearm and contralateral free radial forearm flaps are more reliable than the posterior interosseous flap for the coverage of moderate-sized defects of dorsal or palmar hand and wrist defects following trauma or tumor resection [32]. Ultimately, the choice of flap depends on the surgeon’s experience and preference for a particular technique.

In this report, we present a rare case of a phalangeal enchondroma that demonstrated malignant transformation into chondrosarcoma. Following resection of the fourth ray, recurrence was observed despite wide-margin resection being performed, leading to partial amputation of the ulnar ray of the hand. Although chondrosarcomas rarely metastasize, local aggressiveness has the potential to result in recurrence; therefore, clinical and radiological follow-up must be performed. There is no consensus regarding the management of chondrosarcomas of the hand, and large soft-tissue defects at this site remain challenging to tackle. Based on our experience, we suggest that the posterior interosseous artery flap remains an effective option to cover ulnar soft tissue defects following transcarpal amputation in cases of chondrosarcoma. Finally, it is recommended that such cases are approached by multidisciplinary teams comprising of orthopedicians, pathologists, and oncology specialists.

### Ethical approval

Written informed consent was obtained from the patient to participate in this study for the publication of this case report and accompanying images. A copy of the written consent form is available for review on request.
